# Metabolic adaptation of glucose-deprived macrophages involves partial gluconeogenesis

**DOI:** 10.1073/pnas.2419568122

**Published:** 2025-10-29

**Authors:** Katharina Schindlmaier, Theresa Haitzmann, Visnja Bubalo, Barbara Konrad, Joseph Jelwan, Gabriele Bluemel, Sonja Rittchen, Vanessa Jäger, Michael A. Dengler, Luka Brcic, Jörg Lindenmann, Leigh M. Marsh, Thomas O. Eichmann, Alexander Kirchmair, Zlatko Trajanoski, Julia Kargl, Cristina Muñoz-Pinedo, Katharina Leithner

**Affiliations:** ^a^Division of Pharmacology, Otto Loewi Research Center, Medical University of Graz, Graz 8010, Austria; ^b^Division of Pulmonology, Department of Internal Medicine, Medical University of Graz, Graz 8036, Austria; ^c^Department of Biosciences and Medical Biology, Bioanalytical Research Labs, University of Salzburg, Salzburg 5020, Austria; ^d^Division of Immunology, Otto Loewi Research Center, Medical University of Graz, Graz 8010, Austria; ^e^Division of Oncology, Department of Internal Medicine, Medical University of Graz, Graz 8036, Austria; ^f^Diagnostic and Research Institute of Pathology, Diagnostic and Research Center for Molecular Biomedicine, Medical University of Graz, Graz 8010, Austria; ^g^Lung Research Cluster, Medical University of Graz, Graz 8010, Austria; ^h^Division of Thoracic and Hyperbaric Surgery, Medical University of Graz, Graz 8036, Austria; ^i^Otto Loewi Research Center, Medical University of Graz, Graz 8010, Austria; ^j^Ludwig Boltzmann Institute for Lung Vascular Research, Graz 8010, Austria; ^k^BioTechMed-Graz, Graz 8010, Austria; ^l^Core Facility Mass Spectrometry, Medical University of Graz, Graz 8010, Austria; ^m^Biocenter, Institute of Bioinformatics, Medical University of Innsbruck, Innsbruck 6020, Austria; ^n^Preclinical and Experimental Research in Thoracic Tumors, Bellvitge Biomedical Research Institute, L’Hospitalet 08908, Spain

**Keywords:** macrophages, metabolism, partial gluconeogenesis, glycolysis, glucose deprivation

## Abstract

Macrophages are recruited to sites of infection contributing to the killing of bacteria, but also to malignant tumors, where they promote angiogenesis and suppress antitumor immune responses. The metabolic microenvironment in tumors is frequently depleted of important nutrients such as glucose. Here, we investigated metabolic adaptation strategies of macrophages to glucose deprivation using stable isotopic tracing. Lactate production was decreased, potentially indicating a reduction of glycolysis. In contrast, the contribution of glutamine to the tricarboxylic acid cycle via α-ketoglutarate and reductive carboxylation were increased. Moreover, gluconeogenesis, the reverse pathway of glycolysis, was activated in glucose-deprived macrophages, proceeding partially to the generation of glycolytic intermediates and glycerol-3-phosphate. The partial gluconeogenesis pathway was abrogated in human and murine macrophages lacking the initial gluconeogenic enzyme phosphoenolpyruvate carboxykinase (PCK2, mitochondrial isoform). Partial gluconeogenesis was higher in anti-inflammatory, interleukin-4-stimulated compared to proinflammatory, interferon-γ/lipopolysaccharide-stimulated macrophages. Single-cell analysis and immunostaining revealed expression of PCK2 in macrophages from both lung cancer and normal lung. Low glucose conditions only partially modulated macrophage phenotypes, leading to reduced CD80 surface marker levels in proinflammatory, and enhanced vascular endothelial growth factor expression in anti-inflammatory macrophages. Our study reveals partial gluconeogenesis in glucose-deprived macrophages and shows that this versatile type of immune cells exhibits remarkable metabolic flexibility.

## Significance

Macrophages are versatile immune cells which utilize glucose and other nutrients to fuel their metabolism. Mechanisms of adaptation of macrophages to a limited glucose supply, as present in the tumor microenvironment, are poorly understood. Using stable isotopic tracers, we found that upon glucose deprivation, macrophages reduce lactate production and enhance the usage of glutamine. Interestingly, initial steps of gluconeogenesis, the reverse pathway of glycolysis, were activated, generating cellular intermediates. Glucose deprivation only partially modulated functions of pro- or anti-inflammatory macrophages. The initial gluconeogenesis enzyme, phosphoenolpyruvate carboxykinase, was consistently expressed in human lung and lung cancer macrophages, suggesting its relevance in macrophages in vivo. Our findings show extensive metabolic flexibility of macrophages, involving the activation of partial gluconeogenesis upon glucose deprivation.

Macrophages are part of the innate immune system and are located in almost all tissues of the body. Main macrophage functions are first-line defense via phagocytosis, activation of adaptive immunity, initiation of inflammation, as well as wound healing and tissue repair (1–3). Macrophages may face low concentrations of glucose at sites of inflammation (4) and at different anatomic sites, e.g., in the alveolar space (5). Moreover, the microenvironment of solid tumors often shows reduced levels of glucose (6–8), since the tumor vasculature is frequently abnormal and the perfusion is poor (9, 10). Thus, cancer cells, but also the accompanying macrophages, need to adapt to a highly variable nutrient supply (11). Macrophages show functional and phenotypic heterogeneity, with M1- and M2-like macrophages being two main and very distinct phenotypes (1–3). Classical activation via interferon-gamma (IFNγ) or lipopolysaccharide (LPS) shapes proinflammatory, cytotoxic, and antitumorigenic M1-like macrophages. Alternative activation by interleukin (IL)-4 or IL-13 leads to an anti-inflammatory and protumorigenic M2-like macrophage phenotype, associated with wound healing and tissue repair (1–3).

It is known that the metabolism of macrophages differs across subpopulations of macrophages (12–14). Classically activated, M1-like macrophages have been shown to be “Warburg-like,” highly glycolytic, with increased glucose uptake via solute carrier family 2 member 1 (SLC2A1, GLUT1), and alterations in the tricarboxylic acid (TCA) cycle (15–17). On the other hand, alternatively activated M2-like macrophages have been reported to rely more on TCA cycle metabolism and oxidative phosphorylation (OXPHOS) for energy supply (18, 19). Interestingly, myeloid cells within tumors (predominantly macrophages), not the cancer cells, have been found to be the prime consumers of glucose in a murine colorectal cancer model (20). It is poorly understood how macrophages adapt to the glucose-poor microenvironment in solid tumors and how this adaptation influences their phenotype and functions.

Activation of a partial, truncated form of gluconeogenesis was previously described by us and others to mediate adaptation to glucose deprivation in cancer cells of different origins (21–25). The initial step of gluconeogenesis, the conversion of the TCA cycle intermediate oxaloacetate (OAA) to phosphoenolpyruvate (PEP), is mediated by one of the two isoforms of phosphoenolpyruvate carboxykinase (PEPCK), PCK1, the cytoplasmic isoform, or PCK2, the mitochondrial isoform (26). Dong et al. found PCK2 being upregulated in Kupffer cells, resident macrophages in the liver, upon proinflammatory stimulation, and PCK2 overexpression led to an increase of inflammatory markers (27). Ko et al. described that myeloid-specific deletion of PCK1 enhanced lactate production and promoted a more proinflammatory phenotype (28). Glucose formation from labeled non-carbohydrate precursors (i.e., full gluconeogenesis) was not observed in the macrophages, and glycerol synthesis (glyceroneogenesis, partial gluconeogenesis) was very low; however, gluconeogenesis was assessed only in high (10 mM) glucose-containing medium in that study (28). Fructose-6-phosphate and fructose-1,6-biphosphate were found to be ^13^C-labeled from ^13^C_5_-glutamine by Fan et al. in human monocyte-derived macrophages (MDMs), suggesting gluconeogenesis to the level of sugar-phosphates, which was increased by treatment with yeast-derived whole glucan particles (29). Very recently, Jeroundi et al. demonstrated that macrophages produced stable isotope (^13^C)-labeled glycogen from ^13^C-labeled noncarbohydrate precursors, indicative of glyconeogenesis (30). While these studies showed the capacity of macrophages to perform at least several steps of gluconeogenesis, the role of partial gluconeogenesis in the metabolic adaptation of macrophages to variable glucose conditions is still unclear.

Here, we show that macrophages activate partial gluconeogenesis to generate lower glycolytic intermediates required for anabolic functions, such as glycerol-3-phosphate, under glucose deprivation. Moreover, we show that low glucose conditions reduce lactate production and enhance the contribution of glutamine to the TCA cycle. Production of key inflammatory cytokines, however, was unchanged by low glucose conditions, suggesting a high metabolic flexibility with preserved macrophage functions.

## Results

### Lactate Production Is Reduced in MDMs under Low Glucose Conditions.

**Fig. 1.**
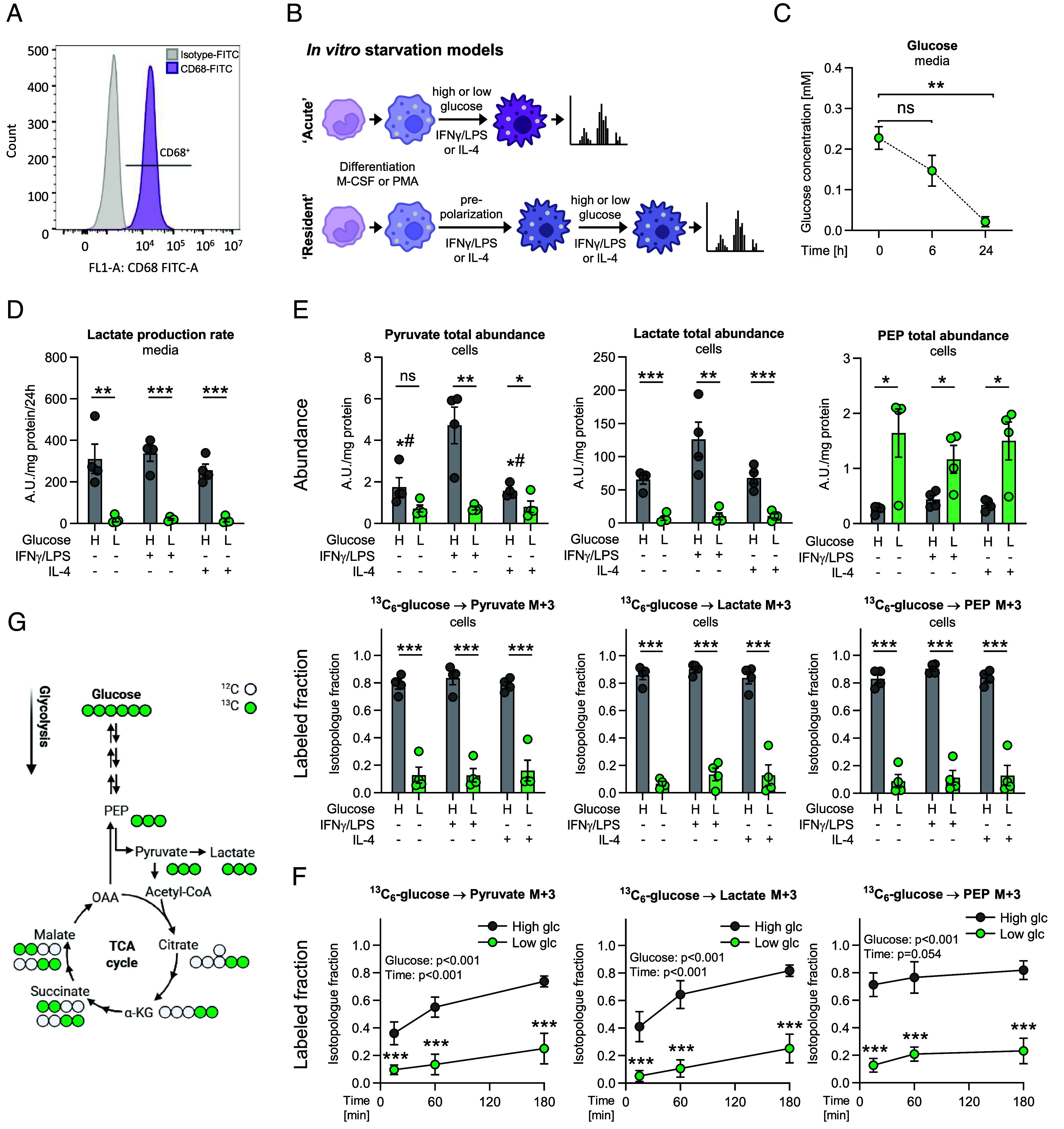
Modulation of metabolism in MDMs by glucose deprivation. (*A*) Representative histogram showing CD68 macrophage marker expression on MDMs compared to isotype control determined by flow cytometry. (*B*) Models of macrophages facing glucose deprivation acutely during (pink) or after (purple) polarization. (*C*) Glucose concentrations in “low glucose” media supernatants. (*D* and *E*) MDMs were cultured in medium containing 10% dialyzed FCS and supplemented with high (H, 10 mM) or low (L, 0.2 mM) levels of ^13^C_6_-glucose. Simultaneously, MDMs were treated with IFNγ/LPS, IL-4, or without stimuli for 24 h (acute model). (*D*) Rate of lactate release to the media normalized to total protein. (*E*) Total abundance (*Top*) of glycolytic intermediates and relative enrichment (*Bottom*, “labeled fraction”) of the fully labeled isotopologues (denoted as M + 3, carrying ^13^C in all three carbon positions) in the cells. (*F*) Time course of labeling fractions in unpolarized MDMs acutely treated with ^13^C_6_-glucose for 15, 60, or 180 min. (*C*–*E*) Data are shown as mean ± SEM from four independent experiments. Group comparisons were performed using unpaired Student’s *t* tests. (*F*) Data are shown as mean ± SEM from four independent experiments. Results from two-way ANOVA assessing the impact of time and glucose are indicated in the graphs, and post hoc analysis for individual time points was performed using Sidak’s test. *P < 0.05, **P < 0.01, ***P < 0.001, n.s., not significant. # vs. IFNγ/LPS-treated cells. (*G*) Labeling patterns of glycolytic and TCA cycle intermediates in MDMs receiving ^13^C_6_-labeled glucose. PEP, phosphoenolpyruvate; OAA, oxaloacetate; α-KG, α-ketoglutarate; glc, glucose.

First, we analyzed metabolic adaptations in macrophages derived from human peripheral blood mononuclear cells (PBMCs) of healthy donors. Stimulation by macrophage colony-stimulating factor (M-CSF) upon adherence to culture plates led to the expected positivity for the macrophage marker CD68 in MDMs (Fig. 1*A*). We additionally used monocytic THP-1 cells differentiated with phorbol-12-myristate-13-acetate (PMA) according to standard protocols (31). To account for metabolic differences in proinflammatory (M1-like) and anti-inflammatory (M2-like) macrophages, we polarized differentiated MDMs or THP-1 cells using IFNγ and LPS or IL-4, respectively. Thereafter, cells were subjected to physiologically high (10 mM) or low (0.2 mM) glucose conditions for 24 to 48 h. This approach mimics macrophage–microenvironment interactions in prepolarized macrophage populations and is referred to as the “resident” model (Fig. 1*B*). Since macrophages have been shown to very rapidly enhance glycolysis upon LPS stimulation (32), we also employed a model in which IFNγ/LPS or IL-4 were administered simultaneously with glucose deprivation (“acute” model, Fig. 1*B*).

To study metabolism in acutely glucose-deprived macrophages, we utilized ^13^C_6_-glucose at a high (10 mM) or low (0.2 mM) concentration to monitor glycolysis and the contribution of glucose to central carbon metabolism with gas chromatography–mass spectrometry (GC-MS). Glucose was rapidly consumed from the medium under low glucose conditions, as expected, although it was still detectable after 24 h (Fig. 1*C*). Accordingly, we replenished high or low glucose media every 24 h to prevent longer starvation periods. We found that the release of lactate to the medium supernatant was greatly decreased in low glucose conditions in all MDMs subpopulations, unstimulated (naïve, M0-like), IFNγ/LPS stimulated (M1-like), or IL-4 stimulated (M2-like) (Fig. 1*D*). Fig. 1*G* depicts the labeling patterns occurring in cells treated with fully ^13^C-labeled glucose. MDMs treated by glucose deprivation showed significantly reduced levels of pyruvate and lactate (Fig. 1*E*, abundance), as well as less incorporation of glucose-derived carbons into glycolytic intermediates (Fig. 1*E*, labeled fraction and *SI Appendix,* Fig. S1*B*), compared to MDMs receiving activation stimuli in high glucose media. Similarly, the time-dependent increase in fractional enrichment of glycolytic intermediates from ^13^C_6_-glucose was decreased under low glucose conditions (Fig. 1*F*). Total levels of the glycolytic intermediate PEP were increased after 24 h (Fig. 1*E*), while they were rather decreased at early time points (*SI Appendix*, Fig. S1*C*). Thus, the decreased labeling fraction of PEP from ^13^C_6_-glucose under low glucose conditions is not solely due to an increased pool size. Taken together, these results show that glycolysis is likely decreased in MDMs under low glucose conditions.

The contribution of glucose to the TCA cycle intermediates α-ketoglutarate (α-KG), malate, and succinate via pyruvate dehydrogenase was diminished under low glucose conditions. This is shown by the reduced fractions of intermediates carrying two ^13^C atoms, denoted M + 2, which are derived from (labeled) acetyl-CoA (*SI Appendix*, Fig. S1 *A* and *B*). To a small part, glucose entered the TCA cycle via pyruvate carboxylation or malic enzyme, as suggested by low but present citrate M + 3 labeling. Low glucose treatment reduced citrate M + 3 fractions as well (*SI Appendix*, Fig. S1*B*). Total levels of citrate and α-KG, normalized to total protein, were decreased under low glucose; however, malate and succinate were unchanged (*SI Appendix*, Fig. S1*A*). Of note, succinate levels were slightly but insignificantly enhanced in M1-like compared to unstimulated or M2-like macrophages (*SI Appendix*, Fig. S1*A*). Despite the potential reduction of glycolysis, the total abundance of the lower glycolytic intermediate phosphoenolpyruvate (PEP) was even enhanced under low glucose conditions, indicating a possible alternative carbon source for PEP under glucose deprivation (Fig. 1*E*).

### Glucose Deprivation Activates Partial Gluconeogenesis in Macrophages, Along with Enhanced Glutamine Contribution to the TCA Cycle.

**Fig. 2.**
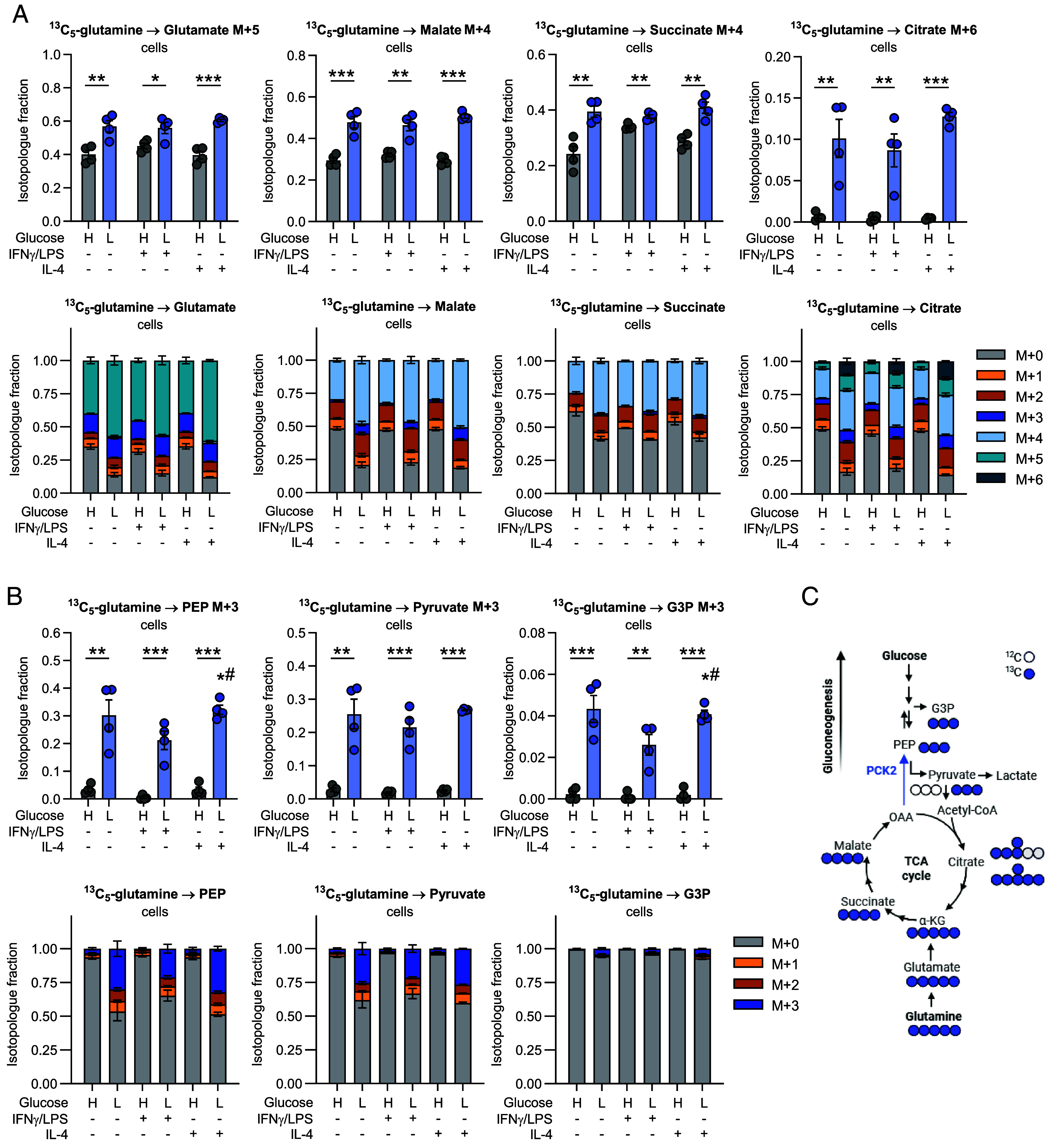
Glucose deprivation induces partial gluconeogenesis in MDMs. (*A* and *B*) MDMs were treated with ^13^C_5_-glutamine as a tracer in medium containing high (H, 10 mM) or low (L, 0.2 mM) levels of glucose and 10% dialyzed FCS simultaneously with IFNγ/LPS or IL-4 or without stimuli for 24 h (acute model). (*A*) Fractions of fully labeled isotopologues of TCA cycle intermediates or (*B*) glycolytic/gluconeogenic intermediates and glycerol-3-phosphate (G3P). (*A* and *B*) Data are shown as mean ± SEM from four independent experiments using MDMs from two different donors. Group comparisons were performed using unpaired Student’s *t* tests. *P < 0.05, **P < 0.01, ***P < 0.001, # vs. IFNγ/LPS-treated cells. (*C*) Labeling patterns of TCA cycle and gluconeogenic/glycolytic intermediates in MDMs receiving ^13^C_5_-labeled glutamine. G3P, glycerol-3-phosphate; PEP, phosphoenolpyruvate; OAA, oxaloacetate; α-KG, α-ketoglutarate; PCK2, phosphoenolpyruvate carboxykinase 2.

We next differentiated macrophages as described above and utilized ^13^C_5_-glutamine as an alternative tracer in high or low glucose conditions simultaneously with the different polarization stimuli, IFNγ/LPS or IL-4. Fig. 2*C* shows the labeling patterns of metabolites derived from fully ^13^C-labeled glutamine. MDMs responded to low glucose levels with an increased contribution of glutamine to the TCA cycle, as we found significantly enhanced ^13^C-labeled fractions of glutamate M + 5, malate M + 4, and succinate M + 4 (Fig. 2*A*). Additionally, full labeling of citrate (M + 6) from the condensation of fully labeled OAA and fully labeled acetyl-CoA, was clearly enhanced by low glucose conditions (Fig. 2*A*). The total abundances of α-KG, malate, and citrate, however, were decreased in low glucose conditions (*SI Appendix*, Fig. S2*A*). Citrate M + 5 labeling and malate M + 3 labeling both were increased under low glucose conditions (Fig. 2 *A*, *Lower* panels and *SI Appendix*, Fig. S2*C*) suggesting that reductive carboxylation contributed to the entry of glutamine carbons to the TCA cycle, especially under glucose deprivation.

Cancer cells facing glucose deprivation have been found to activate initial steps of gluconeogenesis, the reverse pathway of glycolysis as an adaptive mechanism (21, 33). Gluconeogenesis generates glycolytic intermediates from the TCA cycle intermediate OAA, thereby allowing biosynthetic pathways branching from glycolysis to be fueled from noncarbohydrate sources. Neutrophils, which are innate immune cells from the same, myeloid lineage as macrophages, have been shown to use gluconeogenesis to fuel their glycogen stores (34). To clarify whether macrophages use (partial) gluconeogenesis to adapt to low glucose environments, we assessed the conversion of ^13^C_5_-glutamine to the glycolytic intermediate PEP. In the TCA cycle, fully ^13^C-labeled malate gives rise to fully ^13^C-labeled OAA, which in turn is decarboxylated by the gluconeogenic enzyme PEPCK to fully labeled PEP (Fig. 2*C*). Also, malate M + 3, which is converted to OAA M + 3, may potentially contribute to PEP M + 3 labeling, depending on the carbon positions labeled. In fact, up to 30% of PEP (M + 3) were ^13^C labeled under low glucose conditions (Fig. 2*B*). In contrast, only approximately 3% of PEP (M + 3) contained ^13^C from glutamine in high glucose conditions (Fig. 2*B*). PEP was further converted to glycerol-3-phosphate (G3P) via downstream partial gluconeogenesis (Fig. 2*B*). However, labeled glucose was not detected in media supernatants of glucose-deprived macrophages (*SI Appendix,* Fig. S2*B*) showing that gluconeogenesis did not proceed to the generation of glucose but was running partially to the level of glycolytic intermediates.

A similar enhancement of glutamine contribution to the TCA cycle (*SI Appendix*, Fig. S3 *A* and *B*) and activation of partial gluconeogenesis (*SI Appendix*, Fig. S3 *A* and *B*) occurred in the resident model of macrophage–microenvironment interaction. Interestingly, total levels of glycolytic intermediates pyruvate and lactate were likewise decreased by low glucose conditions in the resident model; however, they were similar in unpolarized, M1-like and M2-like macrophages (*SI Appendix*, Fig. S3*C*). Together, our findings indicate that partial gluconeogenesis is robustly activated in MDMs under low glucose conditions.

**Fig. 3.**
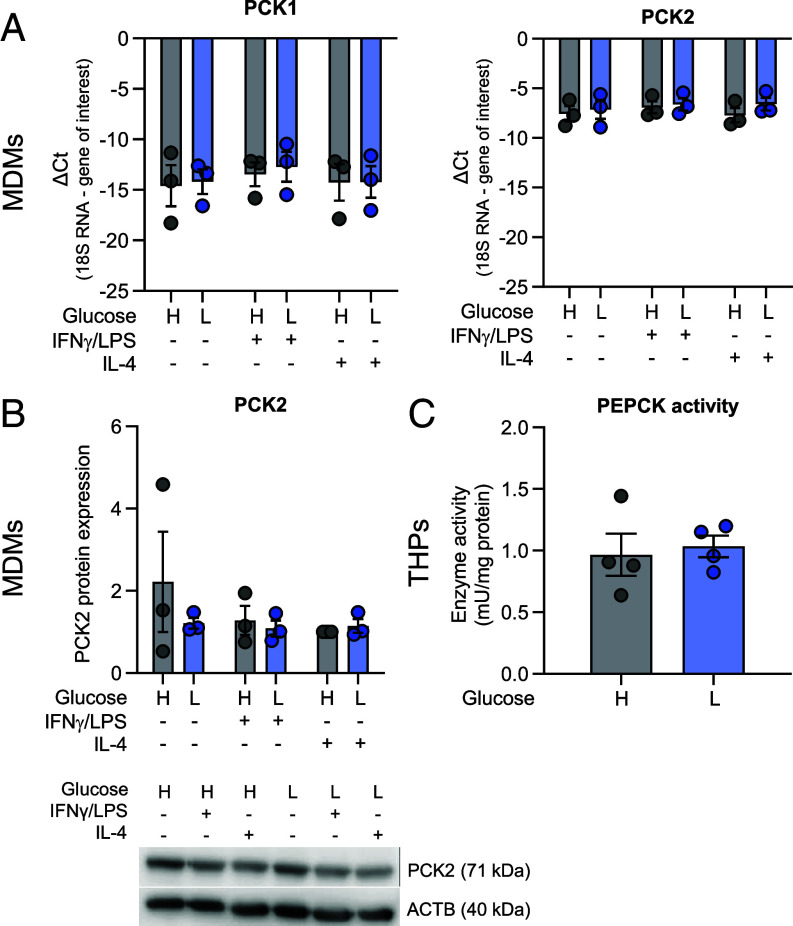
Expression and activity of the gluconeogenesis enzyme phosphoenolpyruvate carboxykinase is independent of polarization and glucose supplementation in MDM. (*A*) PCK1 and PCK2 mRNA levels in differentially prepolarized MDMs (resident model) after high (H) and low (L) glucose treatment for 48 h. (*B*) Representative Western blot of PCK2 and the loading control β-actin (ACTB) in MDMs and quantification of relative intensities. (*C*) PEPCK activity in PMA-differentiated THP-1 cells treated with high or low glucose levels for 48 h. Data are shown as mean ± SEM from three (qPCR, western blot) or four (PEPCK activity) experiments. Group comparisons were performed using unpaired Student’s *t* tests and showed no significant differences.

Next, we sought to clarify whether PCK1 or PCK2 is involved in the initial step of gluconeogenesis. MDMs showed consistent expression of PCK2, on the mRNA and protein level, irrespective of glucose availability or polarization, while PCK1 mRNA abundance was very low in all conditions (Fig. 3 *A* and *B*). THP-1 cells were used to assess PEPCK activity, using a coupled enzymatic assay. Interestingly, PEPCK activity was not modified by glucose (Fig. 3*C*). Thus, the clearly enhanced flux in the direction of gluconeogenesis in low vs. high glucose conditions is caused by changes in substrate and product concentrations rather than modulation of enzyme expression or activity.

### PCK2 Mediates Partial Gluconeogenesis in Human and Mouse Macrophages.

**Fig. 4.**
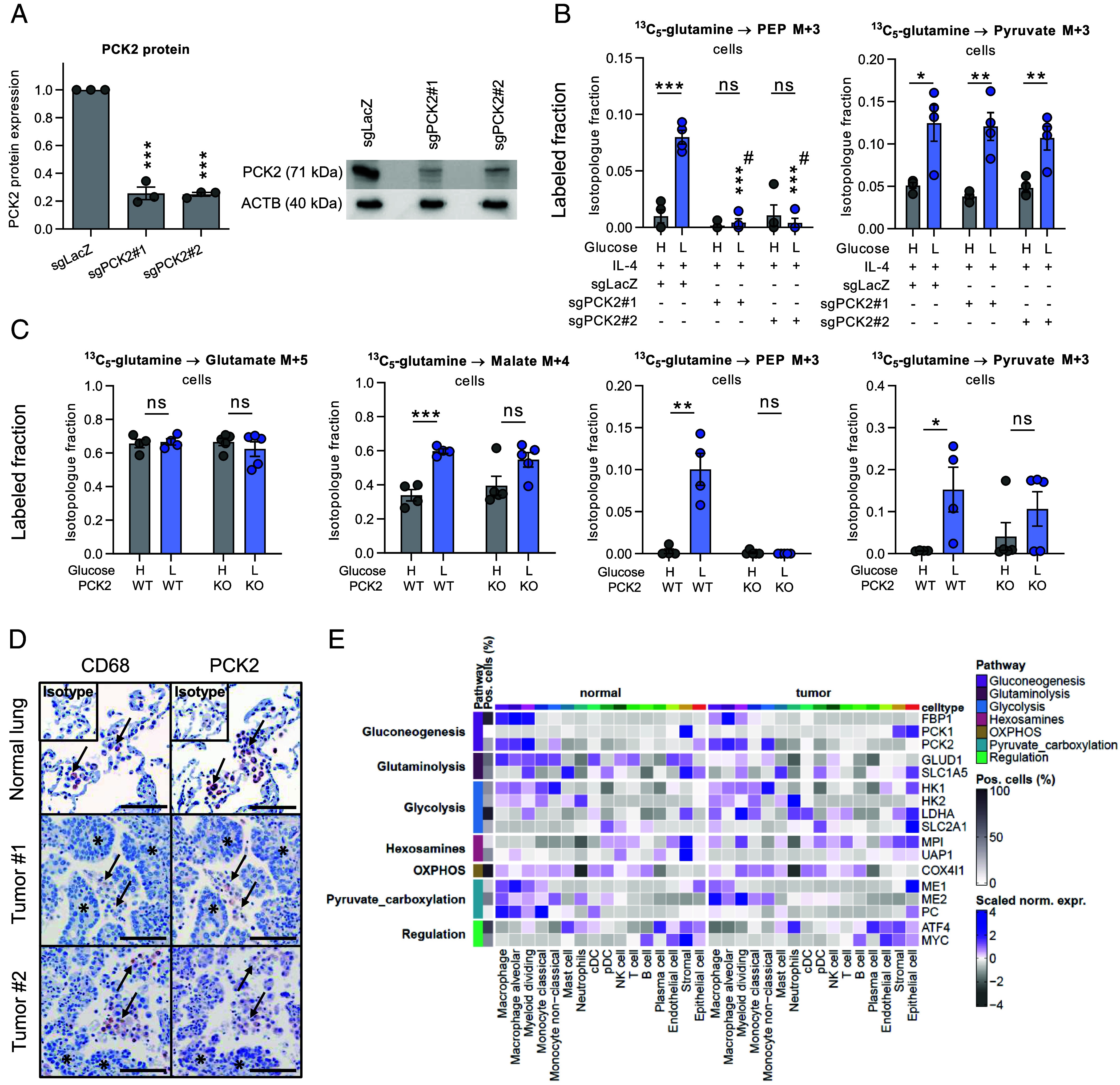
PCK2 mediates partial gluconeogenesis in human and murine macrophages and is expressed in human lung and lung cancer macrophages along with glycolytic genes. (*A*) PCK2 protein levels after CRISPR-Cas9-mediated gene editing with guide RNAs targeting either LacZ (control, sgLacZ) or PCK2 (sgPCK2#1, sgPCK2#2). (*B*) Labeling fractions of PEP and pyruvate in IL-4 prepolarized THP-1 receiving ^13^C_5_-labeled glutamine from four independent experiments. (*C*) Labeling fractions of TCA cycle intermediates, PEP, and pyruvate in peritoneal macrophages from whole body PCK2 WT or KO mice (n = 4 each) treated with ^13^C_5_-labeled glutamine in high (H) or low (L) glucose medium. (*D*) Representative immunohistochemistry stainings of PCK2 and CD68 (macrophage marker) performed on consecutive slides from a normal human lung, a lung squamous cell carcinoma (Tumor#1) or a lung adenocarcinoma (Tumor#2). Arrows: Macrophages, asterisks: tumor cell nests. (Scale bar, 100 µm.) (*E*) Expression of gluconeogenesis, glycolysis, and glutaminolysis genes in different subsets of macrophages from human non-small-cell lung cancers (NSCLCs) or noninvolved normal lung determined by single-cell RNA sequencing (36). (*A–C*) Data are shown as mean ± SEM; Group comparisons were performed using unpaired Student’s *t* test. *P < 0.05, **P < 0.01, ***P < 0.001. ns, not significant.

To confirm the activation of initial steps of gluconeogenesis via PCK2, we created THP-1 PCK2 CRISPR/Cas9 knock-out cells, using two different PCK2 gRNAs (sgPCK2#1, sgPCK2#2), and studied their metabolism in high vs. low glucose conditions using ^13^C_5_-glutamine. Attachment of cells after PMA treatment and expression of the macrophage markers CD68 and CD11b (*SI Appendix*, Fig. S4*A*) confirmed their differentiation into macrophages. PCK2 knockout (KO) efficiency was app. 80% (Fig. 4*A*) in the polyclonal cells, as determined by Western blot. M1- (*SI Appendix*, Fig. S4*C*) and M2-like (*SI Appendix*, Fig. S4*B*) macrophages derived from THP-1 cells transduced with sgLacZ (nontargeting control), sgPCK2#1, or sgPCK2#2 shuttled ^13^C_5_-glutamine toward the TCA cycle in all conditions, and, similar to MDMs, fully ^13^C-labeled malate fractions were enhanced under low glucose treatment. Both M1- and M2-like THP-derived macrophages formed PEP M + 3 from labeled glutamine under low glucose conditions, and the pathway was completely abrogated in PCK2 KO cells (Fig. 4*B* and *SI Appendix*, Fig. S4*C*). These results confirm active cataplerosis (removal of TCA cycle intermediates) (35) and initial steps of gluconeogenesis via PCK2. Interestingly, glutamine conversion to pyruvate was not affected by PCK2 KO (Fig. 4*B* and *SI Appendix*, Fig. S4*D*). This suggests pyruvate formation via an alternative route, such as the malic enzyme pathway (scheme in *SI Appendix*, Fig. S4*E*).

To further confirm the involvement of PCK2 in the adaptive response of macrophages to glucose deprivation, we isolated peritoneal macrophages from PCK2 full-body KO mice. We confirmed that peritoneal macrophages were also capable of switching to partial gluconeogenesis under low glucose conditions which was completely blunted in the PCK2 KO peritoneal macrophages (Fig. 4*C*). These results confirm that PCK2 mediates the initial steps of gluconeogenesis and is involved in the metabolic adaptation in macrophages directly obtained from the peritoneal cavity from mice.

Macrophages residing in the alveolar spaces of the lung as well as tumor-associated macrophages (TAMs) from lung cancers may face glucose deprivation requiring metabolic adaptation. Thus, we examined PCK2 expression in eight samples of human NSCLC and corresponding noninvolved lung and identified macrophages based on their morphology and on CD68 positivity in consecutive serial sections. Macrophages in normal lungs and tumors were consistently positive for PCK2 (Fig. 4*D*). Bronchial epithelial cells and partly also tumor cells were likewise PCK2 positive, as previously shown (37). We analyzed expression of metabolic enzymes in macrophages in a published dataset of NSCLC and normal lung single-cell RNAseq data (36). PCK2 likewise was consistently expressed in different macrophage populations from the normal lung and tumors, while PCK1 expression was barely detectable (Fig. 4*E*). Interestingly, also the downstream gluconeogenic enzyme FBP1 was expressed. All macrophage populations exhibited a strong expression of glycolytic genes and malic enzymes (ME1 and ME2). Moreover, the glutaminolytic enzyme GLUD1 (glutamate dehydrogenase 1) and pyruvate carboxylase, an enzyme interconverting OAA and pyruvate, were clearly expressed in macrophages from normal lung, while their expression was less prominent in tumor macrophages (Fig. 4*E*). Pyruvate carboxylase or malic enzyme could be involved in malate M + 3 and citrate M + 3 labeling observed in ^13^C_6_-glucose treated macrophages under high glucose conditions (*SI Appendix*, Fig. S1*B*). In summary, these data suggest that lung tumor and normal macrophages exhibit vast metabolic flexibility.

### Low Glucose Conditions Reduce the Expression of M1-Like Macrophage Marker CD80, but Do Not Affect Cytokine Production.

**Fig. 5.**
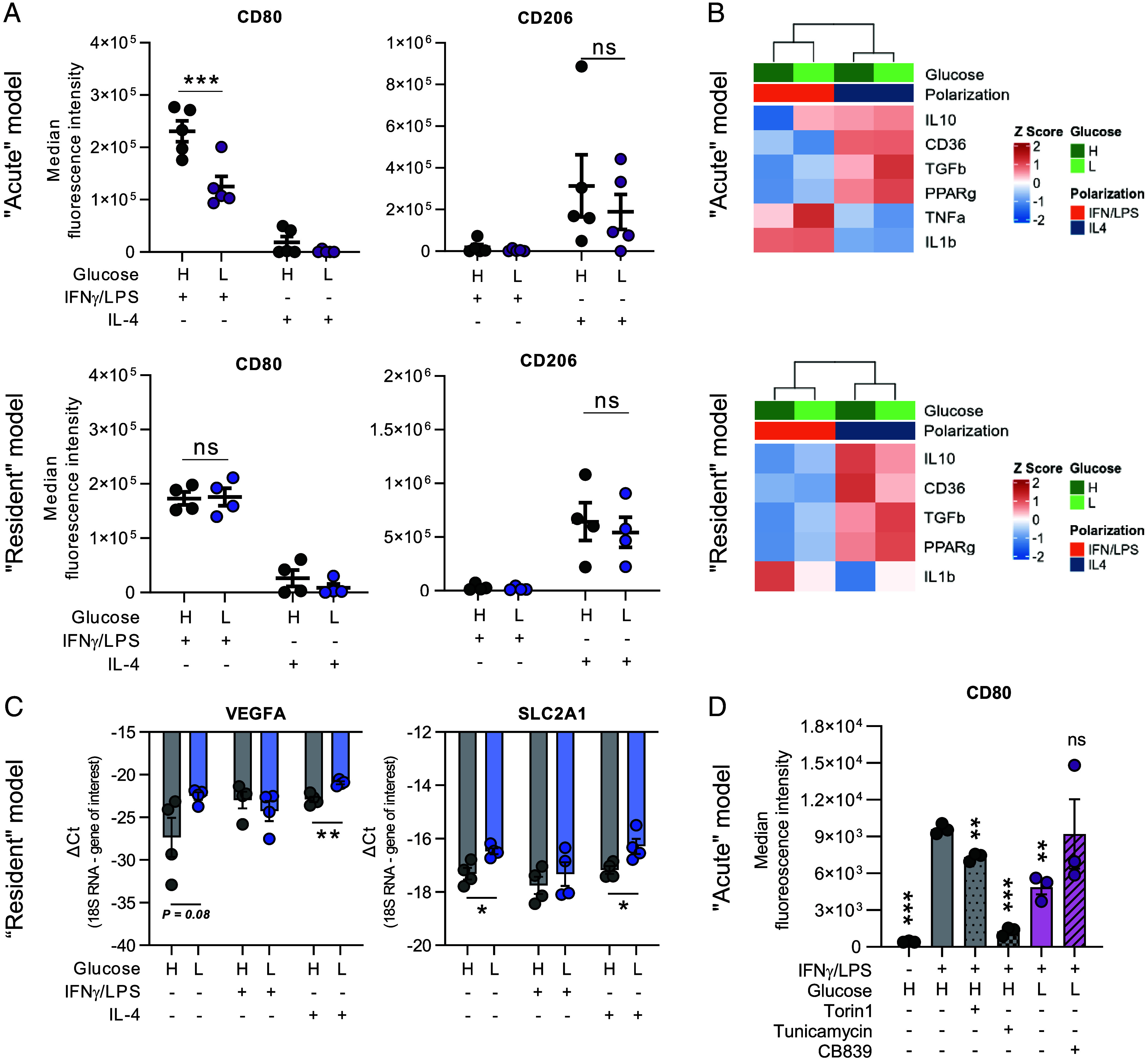
Low glucose impairs expression of M1-like macrophage marker CD80, modulates VEGFA and SLC2A1, but does not affect M1- or M2-related cytokine expression and release. (*A*) CD80 and CD206 in MDMs after high (H) vs. low (L) glucose media determined by flow cytometry. (*B*) Heatmaps showing mean cytokine mRNA levels in MDMs treated with high (H) or low (L) concentrations of glucose, from three (acute model) or four (resident model) independent experiments using MDMs from three different donors in each model. (*C*) Expression of GLUT1 (SLC2A1) and VEGFA mRNA in prepolarized MDMs treated for 48 h with high (H) vs. low (L) glucose, from four independent experiments using MDMs from three different donors. (*D*) CD80 expression was assessed in the acute model of MDMs, treated with high (H) vs. low (L) glucose, Torin1, Tunicamycin, or CB839. Data are shown as mean ± SEM from five experiments/donors (acute model, *A*), four experiments/donors (resident model, *A*), or three experiments/donors (*D*). Heatmaps of scaled expression data, based on hierarchical clustering, were generated using the R package “ggplot2” (R version 4.3.1) (38). Group comparisons were performed using unpaired *t* tests. *P < 0.05, **P < 0.01, ***P < 0.001. (*D*) Significance values are in comparison to the control condition high (H) glucose + IFNγ/LPS.

Finally, we addressed macrophage functions and phenotypes under the different glucose conditions. Surface marker proteins CD80 (M1-like) and CD206 (M2-like) were assessed in MDMs receiving high (10 mM) or low (0.2 mM) glucose treatment, simultaneously with activation stimuli, for 48 h (acute model), or after a prepolarization phase of 48 h (resident model). MDMs receiving simultaneous treatment, but not prepolarized M1-like macrophages, showed significantly decreased expression of CD80 under low glucose conditions (Fig. 5*A*), suggesting that full M1-like polarization requires the presence of glucose, as previously published (32). In contrast, M2-like marker CD206 was unaltered after high vs. low glucose treatment (Fig. 5*A*). Of note, expression of M1- and M2-related cytokines was not significantly altered in low vs. high glucose conditions (Fig. 5*B* showing MDMs). Similarly, cytokine release (*SI Appendix*, Fig. S5 showing THP-1) was not modulated by glucose or PCK2 expression. Interestingly, the expression of vascular endothelial growth factor (VEGFA), a proangiogenic mediator released by TAMs, was significantly enhanced in nonpolarized and M2-like MDMs under low glucose conditions (Fig. 5*C*). Likewise, the main glucose transporter in macrophages, GLUT1 (encoded by *SLC2A1*), was enhanced under low glucose, possibly as an adaptive mechanism (Fig. 5*C*).

Aiming at investigating the mechanism and dependencies of the observed impaired M1-like macrophage activation, we studied the role of the mechanistic target of rapamycin (mTOR) signaling and endoplasmic reticulum (ER) stress, known modulators of proinflammatory macrophages (39–42). Moreover, we blocked glutaminase activity by CB839, given the observed increased glutamine contribution to the TCA cycle. Flow cytometry analyses of MDMs revealed a significant reduction of CD80 expression upon treatment with ER stress inducer Tunicamycin or Torin1, an inhibitor of mTORC1 and mTORC2, under high glucose conditions (Fig. 5*D*). When treating cells with low glucose, glutaminase inhibition rescued CD80 expression in MDMs (Fig. 5*D*). These results suggest that the enhanced glutamine utilization observed under low glucose conditions plays a role in the modulation of M1-like marker CD80. Further, blocking mTOR signaling or N-glycosylation seems to impair formation of M1-like marker CD80, even in glucose-abundant environments.

## Discussion

Our study identified profound changes in central carbon metabolism in macrophages exposed to low glucose conditions including the activation of initial steps of gluconeogenesis and enhancement of glutamine metabolism. Moreover, our findings suggest an important role of glucose during the initial, classical “M1” macrophage activation and expression of CD80, involved in the regulation of T cells (43). We show that partial gluconeogenesis from glutamine is activated by low glucose conditions in macrophages of all polarization states, allowing the generation of glycolytic intermediates from nonglucose precursors. Gluconeogenesis intermediates were shuttled toward the formation of glycerol-3-phosphate, showing that gluconeogenesis proceeds partially, at least to the level of triose-phosphates. Other recent studies found the gluconeogenesis pathway to proceed to the level of glucose-6-phosphate (30) or fructose-6-phosphate (29) in macrophages. Although the PCK2-mediated pathway was robustly activated in our study under low glucose conditions, PCK2 knockout had no effect on the release of classical “M1”- or “M2”-related cytokines in a monocytic cell line, suggesting that macrophages may flexibly adapt to PCK2 inhibition, even in low glucose conditions. Switching, e.g., to the breakdown of glycogen may play a role. As recently reported, glycogen synthesis from glucose or glutamine and glycogen breakdown are dynamically regulated in macrophages (30). The effects of PCK2 in macrophages, however, may be context dependent, and to study the impact of the partial gluconeogenesis on macrophage functions and interactions in vivo, e.g., using cell-specific PCK2 knockout or PEPCK inhibitors would be warranted.

Besides activation of partial gluconeogenesis, low glucose conditions led to a clear reduction of lactate formation and an enhanced contribution of glutamine to the TCA cycle. We observed a significant decrease in the expression of CD80, a marker of proinflammatory macrophages, in low glucose conditions in the MDM model. CD80, as a costimulatory molecule on M1-like macrophages, is crucial for T cell regulation, linking the innate and adaptive immune response (43). Enhanced glutaminolysis and contribution of glutamine to the TCA cycle, as found by our ^13^C tracing approach, may play a role, since the decrease in CD80 expression on M1-like macrophages was reversed by glutaminase inhibition. However, the use of glutamine in alternative pathways such as glutathione synthesis compared to its anaplerotic role in the TCA cycle or the enzymes involved in glutamate conversion to α-KG (oxidation vs. transamination) were not explored in this study. Another further area to study, beyond the scope of this project, could be to address the role of glycerol-phosphate synthesis in macrophages under different polarization stimuli or glucose conditions, with potentially high impact on, e.g., lipid handling, as well as lipid droplet and prostaglandin formation.

The context-dependent effects of glucose levels on macrophage function and metabolism remain poorly understood, yet, they may play an important role in modulating immune and inflammatory responses. Glucose availability may not only change regionally but also systemically according to diet and hormonal regulation. As a limitation of our study, our model of acute vs. resident states of interactions between macrophages and the metabolic TME does not fully recapitulate the situation in different malignant and benign tissues in vivo. The glucose levels used in this study (0.2 mM compared to 10 mM) were chosen arbitrarily to mimic glucose deprivation in vivo; however, they could be controlled only at the beginning of the experiment. Indeed, glucose was rapidly consumed (Fig. 1*C*) and to prevent a prolonged complete lack of glucose, we replaced the media every 24 h. In order to keep glucose levels within a narrow range throughout the experiment, a continuous-flow culture would be needed. However, such a flow device would lead to a very high use of ^13^C tracers; thus, this approach was not feasible.

In our study, we found significantly higher levels of the glycolytic intermediate pyruvate in the proinflammatory macrophages in the acute model, and, by trend, an enhancement of lactate and succinate. These alterations, although subtle, are consistent with the previous literature, showing a preference of M1-like macrophages for glycolysis (15, 16). The reason for relatively mild effects of M1-like polarization on glycolytic intermediates are unknown; however, polarization may lead only to transient increases in glycolysis. Less pronounced increases of lactate or pyruvate in M1-like macrophages in the resident model, as opposed to the acute model, are in line with such an explanation; however, the dynamics and impact of macrophage polarization on glycolysis and/or glycolytic flux should be addressed in future studies. Also, whether upper steps of glycolysis and branching pathways, like the oxidative pentose phosphate pathway, lower glycolysis or the conversion of pyruvate to lactate, are the main metabolic signals for regulating macrophage function is still unknown. The mTOR pathway, for example, has been shown to be directly regulated by the levels of the lower glycolytic intermediate dihydroxyacetone phosphate (44); however, the study was performed in human embryonic kidney cells. As a limitation of our study, we did not assess label enrichment from glucose in more upstream glycolytic intermediates, e.g., using LC–MS. To identify metabolic alterations in glycolysis and branching pathways upon glucose withdrawal, that are translated to the observed CD80 regulation, or to other, reported glucose-dependent phenotypes of macrophages, would be warranted, e.g., using flux analysis (45).

In summary, we found that macrophages likely reduce glycolysis and enhance glutamine use in the TCA cycle, while they activate initial steps of gluconeogenesis via PCK2, suggesting a vast flexibility to fulfill the energetic and biosynthetic needs of macrophages in a changing metabolic microenvironment. Furthermore, a role of PCK2 in different human macrophage populations in vivo is suggested by its robust expression in macrophages from human lungs and lung cancers. The study fosters the understanding of macrophage metabolism and function in different metabolic niches.

## Materials and Methods

Complete experimental methods are described in *SI Appendix*, *Materials and Methods*.

### Human Monocyte–Derived Macrophages from Healthy Donors.

Human monocytes from donors were obtained from the Division of Pharmacology, Medical University of Graz, and characterized as described (46, 47). All experiments involving primary cells or tissues from human subjects were approved by the Review Board of the Medical University of Graz (EK 17-291 ex 05/06, EK 17-215 ex 05/06, and EK 34-003 ex 21/22). All volunteers and patients signed an informed consent.

### CRISPR/Cas9-Mediated Gene Editing and PCK2 Knockout Mice.

CRISPR-Cas9-mediated deletion of PCK2 was performed in THP-1 cells essentially as described (48). PCK2 knockout mice were generated by Cyagen (Santa Clara, CA). Experiments involving PCK2 knockout mice were approved by the Animal Ethics Committee of the Austrian Federal Ministry of Education, Science and Research, and carried out in line with the European Community’s Council Directive. Peritoneal macrophages were harvested from euthanized mice, according to previously published protocols (49), and subjected to stable isotopic labeling ex vivo.

### Stable Isotopic Tracing and Mass Spectrometry.

Cells were treated with the respective treatment media containing 2 mM ^13^C_5_-glutamine or different concentrations of ^13^C_6_-glucose (Sigma-Aldrich, or Cambridge Isotopes, Tewksbury, MA), as tracer for 24 h, either with or without a pretreatment period of 24 h with high (10 mM) or low (0.2 mM) glucose media, containing the ^12^C-analogues. Stable isotopic tracing, sample preparation, gas and liquid chromatography–mass spectrometry procedures (50, 51), peak quantification (52), and natural isotope correction (53) are detailed in *SI Appendix*, *Materials and Methods*.

### Single-Cell RNA Sequencing.

The extended lung cancer atlas (36), available at CELLxGENE, was used to visualize gene expression levels of selected metabolic enzymes. We used the original annotations to calculate pseudobulk gene expression per cell type using the “AverageExpression” function from Seurat v5.1.0 (54), for normal samples (including both healthy normal and tumor-adjacent normal), and tumor samples (including both primary tumors and metastases). The expression values were plotted using the ComplexHeatmap R package v2.18.0 (55). Analyses were performed in R v4.3.2.

### PEPCK Activity.

The PEPCK activity was assessed in the direction of OAA formation essentially as described (56).

## Supplementary Material

Appendix 01 (PDF)

## Data Availability

Metabolomics data have been deposited at Zenodo (https://zenodo.org/records/16085301) (57).
