# Supplementary material for: Metabolic adaptation of glucose-deprived macrophages involves partial gluconeogenesis
Source: Proc Natl Acad Sci U S A. 2025 Oct 29;122(44):e2419568122. doi: 10.1073/pnas.2419568122 (PMC12595420; doi:10.1073/pnas.2419568122)
Supplement: Supplementary file 1 — Appendix 01 (PDF) [file pnas.2419568122.sapp.pdf]

SI Appendix

Materials and Methods

THP-1 Cell Line

THP-1, a human acute monocytic leukemia cell line, was purchased from CLS Cell Lines Service GmbH/Cytion (Eppelheim, DE). Cells were maintained in RPMI 1640 supplemented with 10% fetal bovine serum (FBS), 2 mM L-glutamine, 100 U/mL penicillin and 100 µg/mL streptomycin (RPMI complete), at a cell density of $1x10^6$-$2x10^6$ cells/mL. THP-1 cells were treated with 100 nM phorbol-12-myristate-13-acetate (PMA, Sigma-Aldrich, St. Louis, MO, USA) for 48-72 hours to obtain THP-1-derived, differentiated macrophages (THP-DMs), followed by 24 hours recovery in RPMI complete. For polarization, THP-DMs were cultured in normal growth medium (naïve/M0-like) or supplemented with 100 ng/mL lipopolysaccharide (LPS, Sigma-Aldrich) and 20 ng/mL interferon-gamma (IFNγ, Immunotools, Friesoythe, DE) for M1-like, or 20 ng/mL interleukin 4 (IL-4, Immunotools) for M2-like macrophage activation, for 48-72 hours.

Human Monocyte-Derived Macrophages

Human peripheral blood mononuclear cells (PBMC) were isolated from citrated blood by density gradient sedimentation at the Division of Pharmacology, Medical University of Graz, as previously described (1, 2). PBMCs were plated in adhesion medium (Roswell Park Memorial Institute (RPMI) 1640 medium (Gibco, Waltham, MA, USA) supplemented with 2 mM L-glutamine (Gibco), antibiotics (100 U/mL penicillin and 100 µg/mL streptomycin, Gibco), sodium pyruvate (Sigma-Aldrich), non-essential amino acids (Sigma-Aldrich), Hepes (Sigma-Aldrich), and 5% human serum (Sigma-Aldrich)) for 1.5 hours. After adhesion, medium was changed to differentiation medium (RPMI 1640 containing 2 mM L-glutamine, antibiotics, and 10% FBS (Bio West, Nuaillé, FR), and 20 ng/mL human macrophage colony stimulating factor (M-CSF, PreproTech, Waltham, MA, USA), and cells were cultured for six to eight days. For polarization, MDMs were cultured in normal growth medium (naïve/M0-like) or supplemented with 100 ng/mL lipopolysaccharide (LPS, Sigma-Aldrich) and

20 ng/mL interferon-gamma (IFNγ, Immunotools) for M1-like, or 20 ng/mL interleukin-4 (IL-4, Immunotools) for M2-like macrophage activation, for 48-72 hours.

Mouse Peritoneal Macrophage Isolation

PCK2 wild type or knockout mice were identified by genotyping PCR, after isolation of DNA from tail tips, according to standard protocols. Peritoneal macrophages where isolated from PCK2 wild type or knockout mice, according to established protocols of the Department of Pharmacology, Medical University of Graz, similar to published protocols (3). Briefly, mice were anesthetized with isoflurane, before euthanization via cervical dislocation. Macrophages were isolated by injection of isolation buffer (phosphate buffered saline, PBS, containing 0.1 M EDTA) into the peritoneal cavity. Cells were counted with trypan blue (1:1) on a TC20 automated cell counter (BioRad, Hercules, CA). After isolation, mouse intraperitoneal macrophages were cultured in differentiation media (as described above), additionally supplemented with sodium pyruvate (Sigma-Aldrich), non-essential amino acids (Sigma-Aldrich), Hepes (Sigma-Aldrich), 5% human serum (Sigma-Aldrich), and 100 ng/mL murine M-CSF (Preprotech), for one day before flow cytometry analysis or subsequent low glucose treatment and stable isotopic tracing and GC-MS. After adherence to cell culture plates, identification of the isolated cells as macrophages was done by flow cytometry analysis of classical macrophage marker CD11b, resulting in 92.43% ± 1.19% (mean ± SD) cells positive for CD11b.

PCK2 CRISPR/Cas9 Knockout

THP-1 PCK2 CRISPR/Cas9 knock out cells were generated in collaboration with M.A.D. essentially as described (4). Briefly, THP-1 cells were transduced with a pFUCas9mCherry vector for constitutive expression of the Cas9 protein as well as a doxycycline-inducible pFgh1tUTG lentiviral vector carrying sgRNA targeting LacZ (sgLacZ, control cells) or PCK2 (sgPCK2#1 or sgPCK2#2, PCK2 knockout cells). sgRNA sequences are listed in Table S 4. Transfected THP-1 cells were confirmed by analysis of GFP and mCherry fluorescent proteins. Knockout of PCK2 was induced by adding 1 µg/mL of doxycycline and confirmed by Western blot. Experiments were conducted with polyclonal THP-1 LacZ control cells and two knock out cell lines, THP-1 PCK2 KO#1 and THP-1

PCK2 KO#2, generated with two different guide RNAs, sgPCK2#1 and sgPCK2#2, with a knockout efficiency of more than 80% (Western blot analysis).

Low Glucose Treatments

PBMCs were plated onto 12-well plates at a density of 7-9x10^6 cells/well. After adhesion and differentiation, MDMs were washed 2x with PBS before treatment. MDMs were treated with RPMI SILAC without glucose, without glutamine (Gibco), supplemented with 1.15 mM arginine and 0.27 mM lysine, 2 mM L-glutamine, 10% dialyzed FBS (Gibco), 0.2 or 10 mM glucose, and antibiotics. Polarization stimuli were added as indicated. THP-1 cells were plated onto 6-well or 12-well plates at a density of 1-2x10^6 cells/well, in normal growth media supplemented with 100 nM PMA for 48-72 hours. After adhesion, differentiation and polarization, THP-1 derived macrophages were washed 2x with PBS before treatment. Cells were treated with RPMI SILAC, supplemented arginine/lysine, 2 mM L-glutamine, 10% dialyzed FBS, 0.2 or 10 mM glucose, with or without polarization stimuli, as indicated. Glucose deprivation was applied for 24 or 48 hours with media change after 24 hours. Macrophages received low glucose treatments either along with polarization stimuli (simultaneous treatment), or after a pre-polarization phase (consecutive treatment) of 72 hours, after washing with PBS two times.

Glucose Assay

Cells were subjected to low (0.2 mM) glucose treatments and 50 µL of supernatant media were collected at start of the treatment, after six and after 24 hours. Glucose concentration in the supernatant media was determined with the fluorometric assay of the 'Glucose Assay Kit' (Abcam, Cambridge, UK) according to the manufacturer's protocol. The assay was performed in black 96-well plates with clear optical bottom (Thermo Fisher Scientific, Waltham, MA).

Stable Isotopic Tracing

Cells were pre-treated with polarization agents with or without glucose deprivation as described above. Thereafter, 2 mM $^{13}C_5$-glutamine or different concentrations of $^{13}C_6$-glucose (both Cambridge

Isotopes, Tewksbury, MA or Sigma-Aldrich), were used as tracers for 24 hours, either with or without a pre-treatment period of 24 hours with the respective unlabeled treatment media. Mouse peritoneal macrophages were differentiated as described above for 24 hours, and thereafter treated with high or low glucose media containing 10% dialyzed FBS for 24 hours, followed by $^{13}C_5$-glutamine tracing in high or low glucose medium for additional 24 hours.

Gas Chromatography-Mass Spectrometry (GC-MS)

Metabolite extraction from samples was performed according to previously published protocols (5). After treatment and stable isotopic tracing, supernatant media was collected, centrifuged (400 g, 5 minutes), transferred to new tubes and frozen on liquid nitrogen. Cells were washed 1x with 0.9% NaCl (Fresenius, Bad Homburg, DE) and immediately frozen on liquid nitrogen. As extraction solvent (ice-cold) 62.5% methanol (MeOH, UHPLC, mass spectrometry grade, Sigma-Aldrich) in H_2O, containing norvaline as internal standard (Sigma-Aldrich), was used. For metabolite extraction, cells were scraped on ice with 300 µL (containing 0.18 µg norvaline per sample; 12-well plates) or 640 µL (containing 0.375 µg norvaline per sample; 6-well plates) of extraction solvent per well, and transferred to micro-centrifuge tubes. 20 µL of supernatant media were mixed with 300 µL extraction solvent and norvaline. 400 µL (6-well plate samples) or 187 µL (12-well plate samples and media samples) of ice-cold chloroform (GC-MS grade, Sigma-Aldrich) was added, samples were vortexed thoroughly, and centrifuged (13,000 rpm, 10 minutes, 4°C) for phase separation. The upper phase which contains polar metabolites, was evaporated by vacuum centrifugation overnight. Samples were derivatized with 20 µL methoxyamine (MOX) reagent (2% solution of methoxyamine–hydrogen chloride in pyridine, Thermo Fisher Scientific) at 37°C for 60 minutes. Then, 30 µL N-(tert-butyldimethylsilyl)-N-methyl-trifluoroacetamide with 1% tert-butyldimethylsilyl ethers (MTBSTFA + 1% TBDMS, Thermo Fisher Scientific) was added as silylation reagent. Samples were vortexed, spinned, and incubated at 60°C for 30 minutes. For metabolite separation and detection, the Agilent 78901 GC system coupled with an Agilent 5977A Inert MS system was used. GC was performed using a DB-35ms column and helium as carrier gas with a flow rate of 1 mL/min. In split-less mode, 1 µL sample was injected at an inlet temperature of 270°C. The GC oven was kept at 100°C for 3

minutes and ramped to 300°C with a gradient of 3.5°C/minute. Mass spectrometry was performed at 70 eV and a mass range of 100-650 atomic mass units was measured. El-Maven software (6) was used for peak quantification and isotopologues were corrected for natural abundance with IsoCor (7). Metabolite abundances were normalized to norvaline (internal standard) and the protein content.

Liquid Chromatography – Mass Spectrometry

Media samples (100 µL) were mixed with 400 µL ice-cold methanol under constant shaking (4°C, 10 minutes, 400 rpm). After addition of 300 µL ddH2O and 900 µL methyl tert-butyl ether (MTBE), samples were further incubated under constant shaking (4°C, 20 minutes, 800 rpm). After centrifugation (13,000 rpm, 10 minutes, 4°C), 800 µL of the upper phase were removed and replaced by 800 µL of an artificial upper phase (MTBE/MeOH/ddH_2O, 9/4/4, v/v/v). After additional incubation and centrifugation, as described above, the complete upper phase was removed and 700 µL of the lower phase were collected and dried using a SpeedVac (Thermo Fisher Scientific). Metabolites were resolved in 100 µL 70% acetonitrile (1 mM medronic acid) and used for LC-MS analysis. Two empty extractions (sample-free for background control) were injected before and after sample injections. A glucose reference (for level 1 identification) was injected at the start of the sequence. Chromatographic separation was performed on Vanquish systems (Thermo Fisher Scientific) equipped with an ACQUITY UPLC BEH Amide column (2.1 × 150 mm, 1.7 µm; Waters, Milford, MA), using an 18 minutes gradient (400 µL/min) from 97% solvent A (ACN/ddH_2O, 95/5, v/v; 10 mM NH_4FA, 10 mM NH_3) to 65% solvent B (ddH_2O/ACN, 95/5, v/v; 20 mM NH_4FA, 20 mM NH_3). The column compartment was kept at 40 °C. For ^{13}C analysis an Orbitrap Eclipse Tribrid mass spectrometer (Thermo Fisher Scientific) equipped with a heated electrospray ionization source (OptaMax NG) was used for detection of the metabolites in negative acquisition mode (MS1: m/z 80-900, resolution 120,000, AGC target 3e5, IT auto). Glucose was identified at level 1 via accurate m/z of the [M-H]- ion (< 5 ppm) and comparison of the retention time (rt) and MS2 spectra (separate MS2 analysis of one sample of each group) to the synthetical reference compound. Isotopic peaks of glucose were manually inspected in Freestyle (1.8 SP2) and peak extraction of regarding isotopes

of interest was performed in Skyline (24.1.0.199). Isotopologues were corrected for natural abundance with IsoCor (7).

Quantitative Reverse Transcription Polymerase Chain Reaction (RT-qPCR)

RNA isolation was done using peqGOLD Total RNA Kit (VWR, Radnor, PA, USA) according to the manufacturer’s instructions. 500-1000 ng of isolated RNA was reverse transcribed to cDNA with the qScript cDNA synthesis kit (Quantabio, Beverly, MA, USA) according to the manufacturer’s protocol, in a volume of 20 µL and on the T100^{TM} Thermal Cycler (Bio-Rad, San Francisco, CA, USA). RT-qPCR was done on the BioRad CFX384 Touch Real-Time PCR Detection System (Hercules, CA) using QuantiFast SYBR PCR kit (Qiagen, Hilden, DE). RT-qPCR primers are listed in Table S 1.

Western Blot

Cells were lysed on ice with RIPA buffer (Sigma-Aldrich) and protein concentration was determined with the BCA Protein Assay Kit (Thermo Fisher Scientific) according to the manufacturer’s protocol. Proteins (5-10 µg/lane) were separated using sodium dodecyl sulfate (SDS)-polyacrylamide gel electrophoresis and transferred onto a PVDF membrane (Bio-Rad). Membranes were blocked in 5% milk or bovine serum albumin (BSA) for one hour at RT, and incubated with PCK2 antibody (ab187145, Abcam, Cambridge, UK) 1:2000 in 5% BSA-or β-actin antibody (sc-47778, clone C4, Santa Cruz Biotechnology, Dallas, TX) 1:3000 in 5% milk.

Flow Cytometry Analysis

Cells were harvested with accutase (Sigma-Aldrich) at 37°C for 15-20 minutes, followed by carefully scraping with the plunger of a syringe to remove cells from wells and stained as previously published (1). Reagents and antibodies used for antigen staining are listed in Table S 2 and S 3. Unspecific binding via Fc receptors was blocked with human IgG (100 g/L, 1:100 in PBS, Intratect/Biotest, Dreieich, DE). Flow cytometry was performed on a CytoFLEX S flow cytometer (Beckman Coulter, Brea, CA, USA), with a single-staining strategy using only one fluorophore-conjugated antibody per sample.

ELISA

THP-1 cells were cultured, differentiated, polarized and treated with low glucose media for 24 hours and media supernatants were centrifuged (450 g, 10 minutes, 4°C) and the supernatants were stored at -80°C until analysis. For cytokine analysis 'DuoSet ELISA Kits' (R&D Systems, Minneapolis, MN, USA) were used for IL-1β, TNFα, IL-10 and TGFβ, according to the manufacturer's protocols, before absorption was measured at 450 nm (cytokine concentration) and 560 nm (background correction). Each experimental condition was performed in duplicate wells.

PEPCK Activity

The PEPCK activity assay was performed essentially as described (8). The coupled enzymatic assay measures PEPCK activity in the direction of oxaloacetate formation. Oxaloacetate is further converted to malate by malic dehydrogenase, which in turn leads to the oxidation of nicotinamide adenine dinucleotide (NADH). THP-1 cells were cultured, differentiated and treated with the respective media for 48 hours, with media change after 24 hours. Cells were washed once with PBS and harvested by scraping. After centrifugation (400 g, 5 minutes), cells were lysed in ice-cold isolation buffer (10 mM Hepes pH 7.4, 250 mM sucrose, 1 mM EDTA, 1 mM dithiothreitol) and homogenized by sonication with three 5 second pulses followed by centrifugation at 5000 g for 10 minutes. Protein concentrations in the supernatants were determined using a BCA kit. The reactions were performed in triplicates in 96-well plates (Nunclon Delta Black Microwell SI, Nunc A/S, Roskilde, DK) with a final volume of 200 µL containing 110 mM imidazole-Cl, pH 6.8, 3 mM $MnCl_2$, 13 mM NaF, 10 mM phenylalanine, 1 µM rotenone, 30 mM $NaHCO_3$, 0.15 mM NADH, 6 units/mL malate dehydrogenase, 2 mM phosphoenolpyruvate, and cell homogenate containing 50 µg of protein. Before the experiment the reaction mixture was gassed with 100% CO_2 for 15 minutes. The reaction was initiated with 0.5 mM deoxyguanosine diphosphate (dGDP). The oxidation of NADH by malate dehydrogenase was measured at 355 nm for excitation and 460 nm for emission every 80 seconds for 45 minutes using the CLARIOstar Plus (BMG Labtech, Ortenburg, DE) and MARS Data Analysis Software (BMG Labtech). Control samples lacking HCO_3^-/CO_2 were run simultaneously and this

background NADH consumption was subtracted from the consumption in the complete reaction. One unit of PEPCK activity corresponds to the production of 1 µmol product min^{-1} at 37°C.

Immunohistochemistry

After deparaffinization and antigen retrieval, and Fc-receptor block (Intratect/Biotest, Dreieich, DE, human IgG, 100 g/L, 1:100 in PBS), tissue sections of human lungs and lung cancers were stained with PCK2 rabbit polyclonal antibody (Abcam, ab137580, 1 mg/mL, 1:200 in 0.1% BSA + 0.1% Tween-20 in PBS), or rabbit isotype IgG (Thermo Fisher Scientific, 10500C, 3 mg/mL, 1:600 in 0.1% BSA + 0.1% Tween-20 in PBS), for 90 minutes at RT, or CD68 mouse monoclonal antibody (Santa Cruz, sc-20060, 200 µg/mL, 1:100 in 3% BSA in PBS) or mouse isotype IgG (eBioscience/Invitrogen, 555749, 500 µg/mL, 1:250 in 3% BSA in PBS), for one hour at RT. After protein block with Ultra Vision Protein Block (Epredia, Basel, CH), primary antibody enhancer (Epredia) was applied for 10 minutes and HRP Polymer (Epredia) was added for 15 minutes at RT, before tissue sections were developed with AEC Substrate Kit (Vector lab, Burlingame, CA, USA). Slides were analyzed in consultation with a lung pathologist (Luka Brcic, Medical University of Graz, AT).

Statistics

Data compilation, evaluation and analysis was performed using *GraphPad Prism 9* (Boston, MA), or *IBM SPSS Statistics 29* (Chicago, IL). Statistical evaluation was performed as applicable with two-sided, unpaired Student's t-test or One-Way ANOVA with Dunnett post-hoc analysis. Data are represented as mean ± standard error of the mean (SEM). P-values below 0.05 were considered significant.

Graphs

Figure panels 1*G*, 2*C*, and S4*E* were created with BioRender scientific illustration software.

Supplementary Tables

Table S 1: RT-qPCR primers

Gene	Primer	Sequence (5'-3')
**human PCK2**	forward	CATCCGAAAGCTCCCCAAGTA
	reverse	TGGAAATCAGCTGGGGACATC
**human PCK1**	forward	AAGGAGGATGCCCTGAACCTGAAA
	reverse	TGCACCTTATGGATGGGAAAGGGA
**human TNF-α**	forward	CCCCAGGGACCTCTCTCTAA
	reverse	GCTTGAGGGTTTGCTACAACA
**human TGFB1**	forward	GGAAATTGAGGGCTTTCGCC
	reverse	CCGGTAGTGAACCCGTTGAT
**human IL1B**	forward	CAGAAGTACCTGAGCTCGCC
	reverse	CCTGGAAGGAGCACTTCATCT
**human CD36**	forward	TCCTGCAGAATACCATTTGATCCT
	reverse	TGGTTTCTACAAGCTCTGGTTCTTA
**human PPARG**	forward	AGCCTGCGAAAGCCTTTTGGTG
	reverse	GGCTTCACATTCAGCAAACCTGG
**human IL10**	forward	CAGGGCACCCAGTCTGAGAAC
	reverse	TGGCAACCCAGGTAACCCTTAAA
**human SLC2A1**	forward	TGGCATCAACGCTGTCTTCT
	reverse	AGCCAATGGTGGCATACACA
**human VEGFA**	forward	AACATCACCATGCAGATTATGCG
	reverse	CGTACACGCTCCAGGACTTA
**human ACTB**	forward	ATTGCCGACAGGATGCAGGAA
	reverse	GCTGATCCACATCTGCTGGAA
**human 18S-rRNA**	forward	CTACCACATCCAAGGAAGCA
	reverse	TTTTTCGTCACTACCTCCCCG

Table S 2: Flow cytometry reagents

Reagent	Concentration	Company	Cat. No.
**Fixation/Permeabilization solution**	-	BD Biosciences	554722
**Perm/Wash Buffer (10X)**	-	BD Biosciences	421002
**Zombie Aqua™ Fixable Viability Kit** (viability stain)	1:1000	BioLegend	423107
**Intratect Immunglobulin G** (human Fc block)	1:100	Biotest	-
**TruStain FcX™ PLUS (anti-mouse CD16/32) AB** (murine Fc block)	1:100	BioLegend	156603

Table S 3: Flow cytometry antibodies

Antigen	Antibody	Concentration	Company	Cat. No.
**human CD68**	FITC mouse anti-human CD68	1:25	BioLegend	333806
**human CD80**	PE mouse anti-human CD80	1:10	BD Biosciences	557227
**human CD206**	PE mouse anti-human CD206	1:10	BD Biosciences	555954
**mouse IgG2b**	FITC mouse IgG2b, κ Isotype Control	1:166	BioLegend	400310
**mouse IgG1**	PE mouse IgG1, κ Isotype Control	1:10	BD Biosciences	555749
**human CD11b**	PE-Cy™7 mouse anti-human CD11b	1:166	BioLegend	301322
**mouse IgG1**	PE-Cy™7 mouse IgG1, κ Isotype Control	1:166	BioLegend	406613
**mouse CD11b**	PE-Cy™7 rat anti-mouse CD11b	1:10	BD Biosciences	552850
**rat IgG2b**	PE-Cy™7 rat IgG2b, κ Isotype Control	1:10	BD Biosciences	552849

Table S 4: CRISPR/Cas9 sgRNA sequences

sgRNA	Target sequence for sgRNA (5'-3')
**sgPCK2#1**	TGCGTATTATGACCCGACTG
**sgPCK2#2**	GGCACGAGTAGAGAGCAAGA

Supplementary References

1. S. Rittchen *et al.*, Monocytes and Macrophages Serve as Potent Prostaglandin D(2) Sources during Acute, Non-Allergic Pulmonary Inflammation. *Int J Mol Sci* **22** (2021).

2. A. Hartnell *et al.*, Identification of selective basophil chemoattractants in human nasal polyps as insulin-like growth factor-1 and insulin-like growth factor-2. *J Immunol* **173**, 6448-6457 (2004).

3. A. De Jesus *et al.*, Optimized protocol to isolate primary mouse peritoneal macrophage metabolites. *STAR Protoc* **3**, 101668 (2022).

4. B. J. Aubrey *et al.*, An inducible lentiviral guide RNA platform enables the identification of tumor-essential genes and tumor-promoting mutations in vivo. *Cell Rep* **10**, 1422-1432 (2015).

5. D. Lorendeau *et al.*, Dual loss of succinate dehydrogenase (SDH) and complex I activity is necessary to recapitulate the metabolic phenotype of SDH mutant tumors. *Metab Eng* **43**, 187-197 (2017).

6. S. Agrawal *et al.*, El-MAVEN: A Fast, Robust, and User-Friendly Mass Spectrometry Data Processing Engine for Metabolomics. *Methods Mol Biol* **1978**, 301-321 (2019).

7. P. Millard *et al.*, IsoCor: isotope correction for high-resolution MS labeling experiments. *Bioinformatics* **35**, 4484-4487 (2019).

8. R. Stark *et al.*, Phosphoenolpyruvate cycling via mitochondrial phosphoenolpyruvate carboxykinase links anaplerosis and mitochondrial GTP with insulin secretion. *J Biol Chem* **284**, 26578-26590 (2009).

Supplementary Figure Legends

Supplementary Fig. S1: Glucose deprivation reduces glucose contribution to the tricarboxylic acid (TCA) cycle and TCA cycle metabolite abundance. MDMs were treated with medium containing high (H, 10 mM) or low (L, 0.2 mM) levels of $^{13}C_6$-glucose simultaneously with IFNγ/LPS or IL-4 or without stimuli for 24 hours ('acute' model) or in a time-course experiment to assess shorter time points (15 min, 60 min, or 180 min). (*A*) Total abundance (top) and M+2 labeled fractions (bottom) of TCA cycle intermediates are shown as mean ± SEM from n=4 independent experiments using MDMs from four donors. (*B*) Isotopologue fractions (staggered) of glycolytic and TCA cycle intermediates. Group comparisons were performed using unpaired t-tests. (*C*) Time course of total abundances of glycolytic intermediates pyruvate, lactate and phosphoenolpyruvate (PEP) in unpolarized MDMs acutely treated with high or low levels of $^{13}C_6$-glucose are shown as mean ± SEM from n=3 independent experiments. Results from Two-way ANOVA assessing the impact of time and glucose are indicated in the graphs and post-hoc analysis for individual time points was performed using Sidak's test. *p<0.05, **p<0.01, ***p<0.001; ns, not significant.

Supplementary Fig. S2: Cellular metabolites and medium supernatant glucose in $^{13}C_5$-glutamine treated cells. MDMs were treated with $^{13}C_5$-glutamine as a tracer in medium containing high (H, 10 mM) or low (L, 0.2 mM) levels of glucose simultaneously with IFNγ/LPS or IL-4 or without stimuli for 24 hours ('acute' model). (*A*), Total cellular metabolite abundances and (*C*), citrate M+5 and malate M+3 isotopologe fractions. (*B*) Relative glucose isotopologue abundance in media supernatants measured by LC-MS. Only unlabeled glucose is found after correction for natural abundance. (*A-C*) Data are shown as mean ± SEM from n=4 independent experiments using MDMs from two different donors. Group comparisons were performed using unpaired t-tests. *p<0.05, **p<0.01, ***p<0.001; ns, not significant.

Supplementary Fig. S3: Glucose deprivation induces partial gluconeogenesis in pre-polarized MDMs ('resident' model). (A-C) Non-activated or IFNγ/LPS or IL-4 activated THP-DMs were treated with medium containing 10 mM (H) or 0.2 mM (L) glucose, 10% dialyzed FBS, and 2 mM of glutamine for 24 hours. Thereafter glutamine was replaced with $^{13}C_5$-labeled glutamine for additional 24 hours

(total glucose deprivation phase of 48 hours). (*A*) Relative enrichment of fully labeled isotopologues of TCA cycle intermediates, glycolytic/gluconeogenic intermediate PEP, pyruvate and glycerol-3-phosphate (G3P). (*B*) Isotopologue fractions (staggered) of glycolytic and TCA cycle intermediates. *(C)* Total abundance of lactate and pyruvate. Data are shown as mean ± SEM from n=5 independent experiments using MDMs from four different donors. Group comparisons were performed using unpaired t-tests. *p<0.05, **p<0.01, ***p<0.001, n.s. not significant; # v.s. IFNγ/LPS-treated cells.

Supplementary Fig. S4: Impact of PCK2 knockout on central carbon metabolism. *(A)* Macrophage markers in human PMA-treated or non-treated THP-1 cells. Labeled fractions in PCK2 knockout THP-1 cells (sgPCK2#1, sgPCK2#2) or control cells (sgLacZ) pre-polarized with IL-4 (*B*) or IFNγ/LPS (*C*, *D*) and treated with $^{13}C_5$-glutamine from n=4 independent experiments. *(E)* Labeling patterns from $^{13}C_5$-glutamine entering partial gluconeogenesis. Data are shown as mean ± SEM. Group comparisons were performed by unpaired t-tests. *p<0.05, **p<0.01, ***p<0.001, n.s. not significant; # v.s. IFNγ/LPS-treated cells. G3P, glycerol-3-phosphate; PEP, phosphoenolpyruvate; OAA, oxaloacetate; α-KG, α-ketoglutarate; ME, malic enzyme.

Supplementary Fig. S5: Impact of PCK2 knockout on cytokine secretion to the supernatant media. Secretion of IL-1β, TNFα, IL-10 and TGFβ were determined in macrophages derived from CK2 knockout THP-1 cells (sgPCK2#1, sgPCK2#2) or control cells (sgLacZ), treated with high (H) vs. low (L) glucose media, simultaneously with activation stimuli IFNγ/LPS or IL-4 (‘acute’ model), for 24 hours (M1-related cytokines), or 48 hours (M2-related cytokines). Photometrical determination of cytokine secretion in the supernatant media was performed from n=3 independent experiments. Data are shown as mean ± SEM. Group comparisons were performed by unpaired t-tests and showed no significant differences.

Figure S1

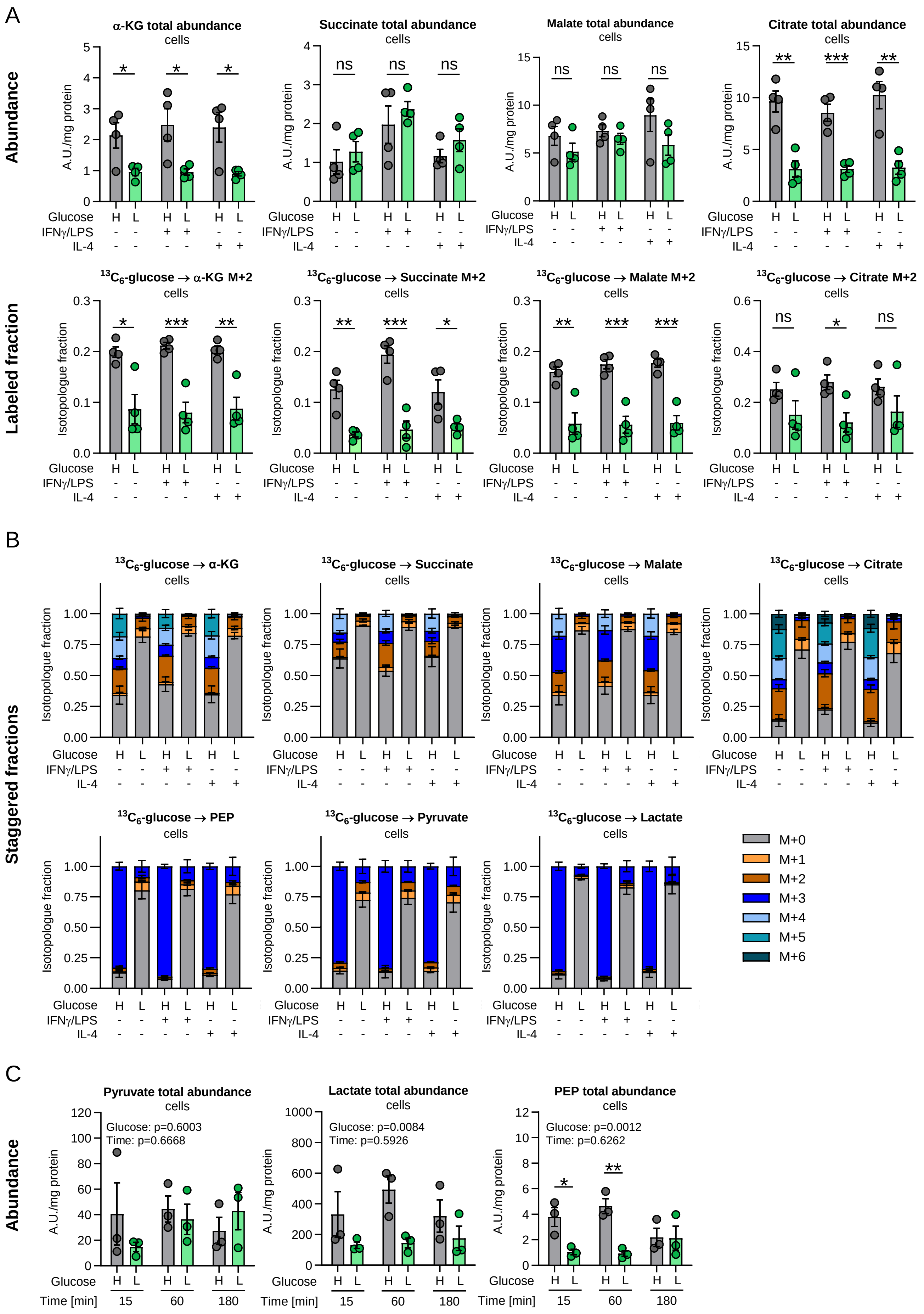
A
Abundance
α-KG total abundance
cells
Succinate total abundance
cells
Malate total abundance
cells
Citrate total abundance
cells
A.U./mg protein
Glucose H L H L H L
IFNγ/LPS - - + + - -
IL-4 - - - - + +
*
**
***
ns
Labeled fraction
13C6-glucose → α-KG M+2
13C6-glucose → Succinate M+2
13C6-glucose → Malate M+2
13C6-glucose → Citrate M+2
Isotopologue fraction
B
Staggered fractions
13C6-glucose → α-KG
13C6-glucose → Succinate
13C6-glucose → Malate
13C6-glucose → Citrate
13C6-glucose → PEP
13C6-glucose → Pyruvate
13C6-glucose → Lactate
M+0
M+1
M+2
M+3
M+4
M+5
M+6
C
Abundance
Pyruvate total abundance
cells
Glucose: p=0.6003
Time: p=0.6668
Lactate total abundance
cells
Glucose: p=0.0084
Time: p=0.5926
PEP total abundance
cells
Glucose: p=0.0012
Time: p=0.6262
Time [min] 15 60 180

Figure S2

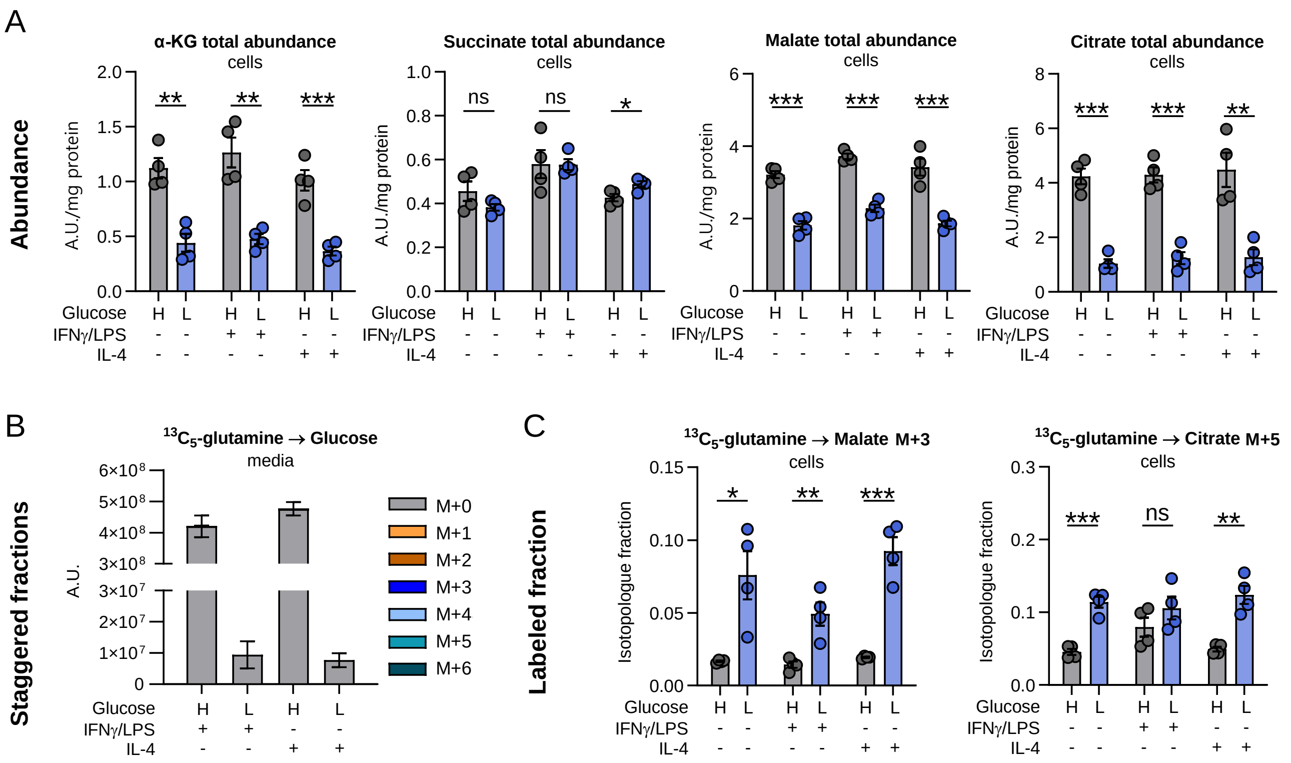
A
Abundance
α-KG total abundance
cells
A.U./mg protein
Succinate total abundance
Malate total abundance
Citrate total abundance
ns
Glucose
IFNγ/LPS
IL-4
B
Staggered fractions
$^{13}C_5$-glutamine → Glucose
media
A.U.
M+0
M+1
M+2
M+3
M+4
M+5
M+6
C
Labeled fraction
$^{13}C_5$-glutamine → Malate M+3
$^{13}C_5$-glutamine → Citrate M+5
Isotopologue fraction

Figure S3

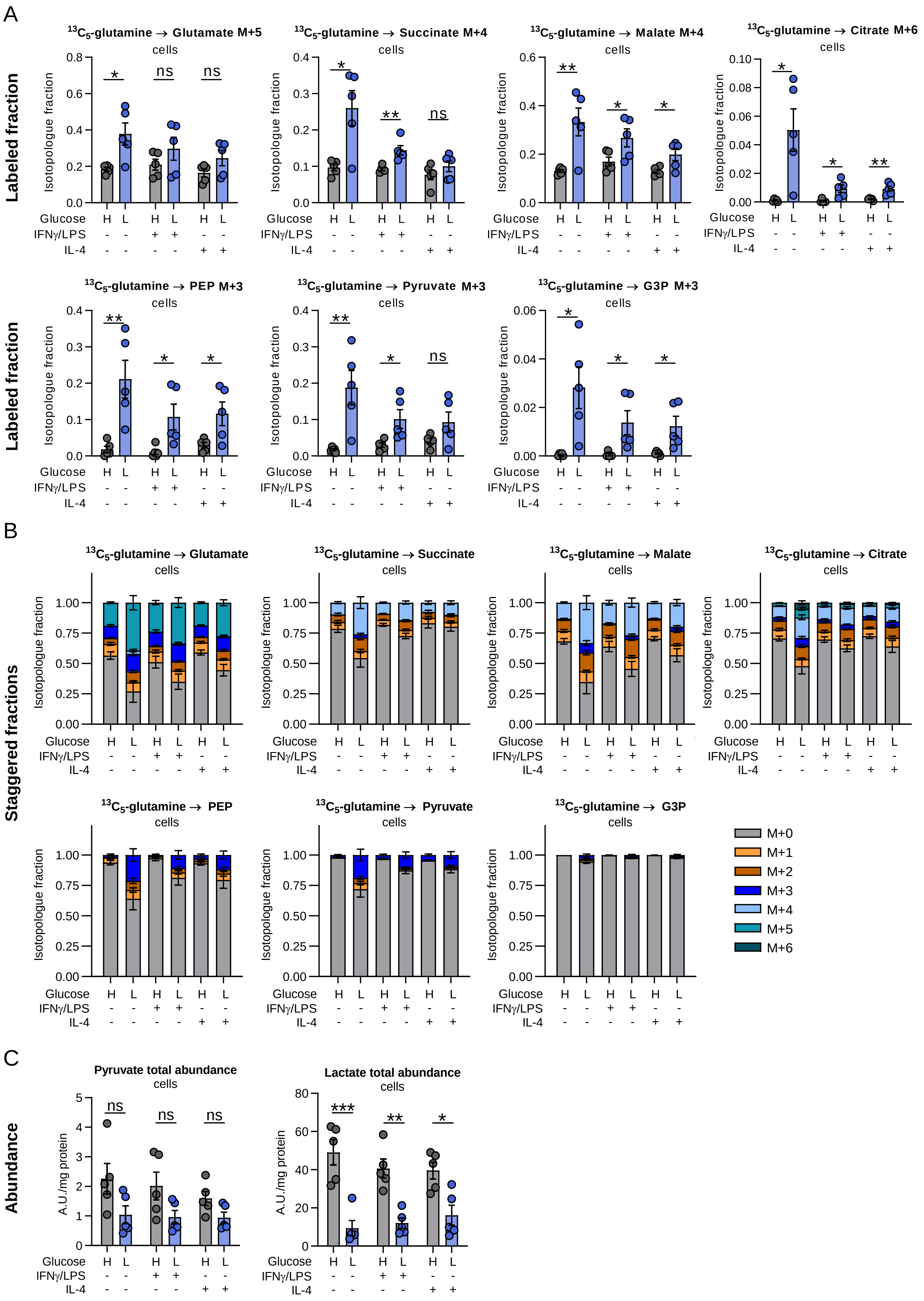
A
Labeled fraction
$^{13}C_5$-glutamine → Glutamate M+5
cells
$^{13}C_5$-glutamine → Succinate M+4
cells
$^{13}C_5$-glutamine → Malate M+4
cells
$^{13}C_5$-glutamine → Citrate M+6
cells
$^{13}C_5$-glutamine → PEP M+3
cells
$^{13}C_5$-glutamine → Pyruvate M+3
cells
$^{13}C_5$-glutamine → G3P M+3
cells
Isotopologue fraction
Glucose
IFNγ/LPS
IL-4
B
Staggered fractions
$^{13}C_5$-glutamine → Glutamate
cells
$^{13}C_5$-glutamine → Succinate
cells
$^{13}C_5$-glutamine → Malate
cells
$^{13}C_5$-glutamine → Citrate
cells
$^{13}C_5$-glutamine → PEP
cells
$^{13}C_5$-glutamine → Pyruvate
cells
$^{13}C_5$-glutamine → G3P
cells
M+0
M+1
M+2
M+3
M+4
M+5
M+6
C
Abundance
Pyruvate total abundance
cells
Lactate total abundance
cells
A.U./mg protein

Figure S4

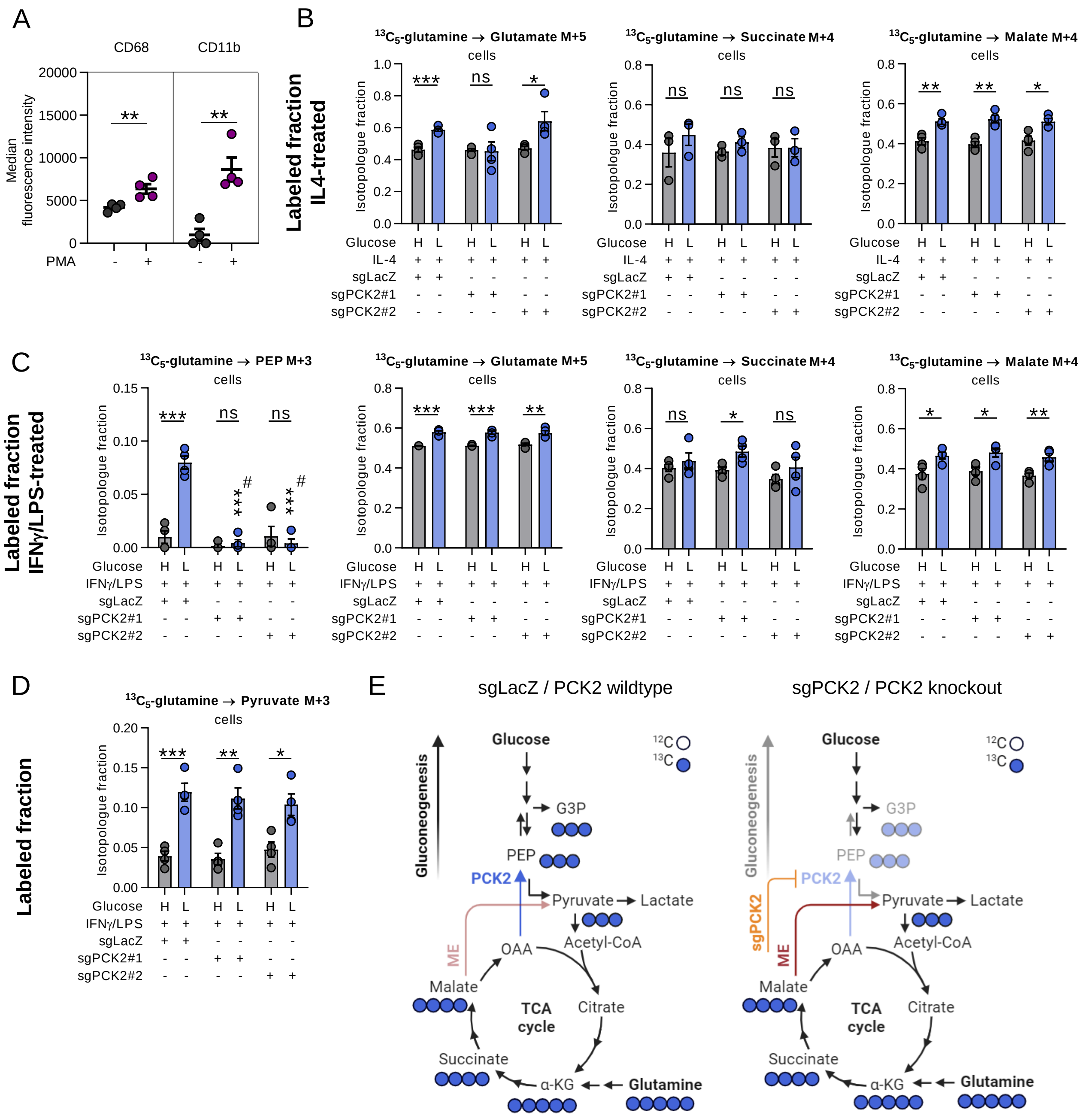
A
CD68
CD11b
Median fluorescence intensity
PMA
B
Labeled fraction IL4-treated
$^{13}C_5$-glutamine → Glutamate M+5 cells
$^{13}C_5$-glutamine → Succinate M+4 cells
$^{13}C_5$-glutamine → Malate M+4 cells
Isotopologue fraction
Glucose
IL-4
sgLacZ
sgPCK2#1
sgPCK2#2
C
Labeled fraction IFNγ/LPS-treated
$^{13}C_5$-glutamine → PEP M+3 cells
IFNγ/LPS
D
Labeled fraction
$^{13}C_5$-glutamine → Pyruvate M+3 cells
E
sgLacZ / PCK2 wildtype
sgPCK2 / PCK2 knockout
Gluconeogenesis
Glucose
G3P
PEP
PCK2
Pyruvate
Lactate
Acetyl-CoA
OAA
ME
Malate
TCA cycle
Citrate
Succinate
α-KG
Glutamine
sgPCK2

Figure S5

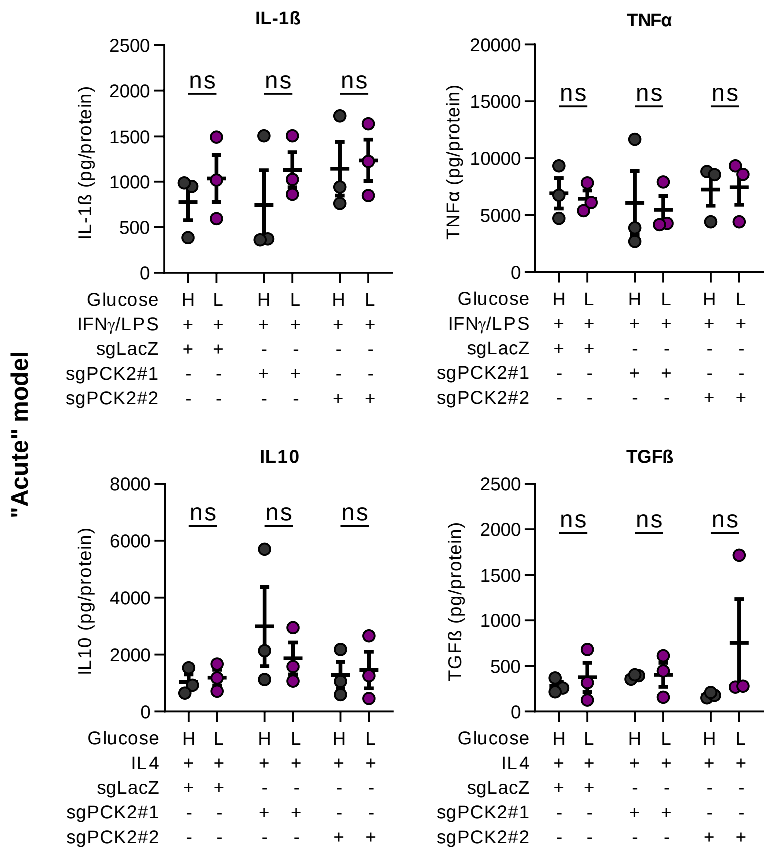

"Acute" model
IL-1ß
IL-1ß (pg/protein)
2500
2000
1500
1000
500
0
ns
ns
ns
TNFα
TNFα (pg/protein)
20000
15000
10000
5000
0
ns
ns
ns
Glucose H L H L H L
IFNγ/LPS + + + + + +
sgLacZ + + - - - -
sgPCK2#1 - - + + - -
sgPCK2#2 - - - - + +
IL10
IL10 (pg/protein)
8000
6000
4000
2000
0
ns
ns
ns
TGFß
TGFß (pg/protein)
2500
2000
1500
1000
500
0
ns
ns
ns
Glucose H L H L H L
IL4 + + + + + +
sgLacZ + + - - - -
sgPCK2#1 - - + + - -
sgPCK2#2 - - - - + +
